# Oxygen-Ozone Therapy for Reducing Pro-Inflammatory Cytokines Serum Levels in Musculoskeletal and Temporomandibular Disorders: A Comprehensive Review

**DOI:** 10.3390/ijms23052528

**Published:** 2022-02-25

**Authors:** Alessandro de Sire, Nicola Marotta, Martina Ferrillo, Francesco Agostini, Cristiano Sconza, Lorenzo Lippi, Stefano Respizzi, Amerigo Giudice, Marco Invernizzi, Antonio Ammendolia

**Affiliations:** 1 Physical Medicine and Rehabilitation Unit, Department of Medical and Surgical Sciences, University of Catanzaro “Magna Graecia”, 88100 Catanzaro, Italy; nicola.marotta@unicz.it (N.M.); ammendolia@unicz.it (A.A.); 2 Department of Health Sciences, University of Catanzaro “Magna Graecia”, 88100 Catanzaro, Italy; martinaferrillo@hotmail.it (M.F.); a.giudice@unicz.it (A.G.); 3 Department of Anatomy, Histology, Forensic Medicine and Orthopedics, Sapienza University, 00185 Rome, Italy; francesco.agostini@uniroma1.it; 4 IRCCS Humanitas Research Center, Via Manzoni 56, 20089 Rozzano, Italy; cristiano.sconza@humanitas.it (C.S.); stefano.respizzi@humanitas.it (S.R.); 5 Department of Biomedical Sciences, Humanitas University, Via Rita Levi Montalcini 4, 20090 Pieve Emanuele, Italy; 6 Physical and Rehabilitative Medicine, Department of Health Sciences, University of Eastern Piedmont “A. Avogadro”, 28100 Novara, Italy; lorenzolippi.mt@gmail.com (L.L.); marco.invernizzi@med.uniupo.it (M.I.); 7 Translational Medicine, Dipartimento Attività Integrate Ricerca e Innovazione (DAIRI), Azienda Ospedaliera SS. Antonio e Biagio e Cesare Arrigo, 15121 Alessandria, Italy

**Keywords:** ozone, oxygen-ozone therapy, musculoskeletal disorders, temporomandibular disorders, pain management, rehabilitation, low back pain, osteoarthritis, inflammation

## Abstract

To date, the application of oxygen-ozone (O_2_O_3_) therapy has significantly increased in the common clinical practice in several pathological conditions. However, beyond the favorable clinical effects, the biochemical effects of O_2_O_3_ are still far from being understood. This comprehensive review aimed at investigating the state of the art about the effects of O_2_O_3_ therapy on pro-inflammatory cytokines serum levels as a modulator of oxidative stress in patients with musculoskeletal and temporomandibular disorders (TMD). The efficacy of O_2_O_3_ therapy could be related to the moderate oxidative stress modulation produced by the interaction of ozone with biological components. More in detail, O_2_O_3_ therapy is widely used as an adjuvant therapeutic option in several pathological conditions characterized by chronic inflammatory processes and immune overactivation. In this context, most musculoskeletal and temporomandibular disorders (TMD) share these two pathophysiological processes. Despite the paucity of in vivo studies, this comprehensive review suggests that O_2_O_3_ therapy might reduce serum levels of interleukin 6 in patients with TMD, low back pain, knee osteoarthritis and rheumatic diseases with a concrete and measurable interaction with the inflammatory pathway. However, to date, further studies are needed to clarify the effects of this promising therapy on inflammatory mediators and their clinical implications.

## 1. Introduction

Ozone gas (O_3_) was discovered in 1840, and its expansion into the medical field has given rise to compelling research in the recent decades to validate its clinical value [1]. Despite some controversies, several papers [2,3,4,5,6,7,8,9,10,11] have proposed relevant medical features, including bactericidal and virucidal effects, inflammatory modulation and circulatory stimulation, with considerable applications in several medical fields such as wound healing, ischemic disorders, infections, and chronic inflammatory conditions such as musculoskeletal disorders.

The function of O_3_ shares similarities with that of a prodrug, as it is modified upon reacting with molecules to develop more active substrates, thus prompting an endogenous cascade of reactions [12]. On the other hand, it is hard to classify O_3_ as merely a prodrug, due to its power to directly interact with phospholipids, lipoproteins, bacteria envelopes and viral capsids. Therefore, O_3_ is considered one of the most powerful oxidizing molecules in nature, although, at high concentrations, it rapidly decomposes into ordinary oxygen [13]. O_3_ rapidly reacts with water and polyunsaturated fatty acids (PUFA) and in human fluids and tissues, producing, respectively, hydrogen peroxide (H_2_O_2)_ and a combination of lipid ozonation products (LOP), mainly composed by 4-HNE (from omega-6 PUFA) and 4-HHE (trans-4 hydroxy-2-hexenal from omega-3 PUFA) [13]. In this context, H_2_O_2_, acting as an ozone messenger, is considered as the fundamental reactive oxygen species (ROS). However, other ROS have been identified as products of ozone reactions, such as superoxide ions and hydroxyl radical (OH^−^) [14]. Hence, given the role of signal transduction, the previous concept that ROS are always harmful has recently been revised and replaced by the latest evidence describing ROS as mediators of the host defense and immune responses [15].

Cytokines are undoubtedly involved in these processes and the proinflammatory cytokines tumor necrosis factor-α (TNF-α), interleukin-1 (IL-1), macrophage migration inhibitory factor (MIF) play a pivotal role [16,17]. Cytokines have been considered encouraging biomarkers and clinical targets in rheumatic and oncologic therapies, but, to date, anti-cytokine-based therapeutic approaches such as the use of anti-TNF antibodies, soluble TNF receptors or IL-1 receptor antagonists have failed to ascertain a clear clinical advantage [18,19]. In addition, some antioxidants and ROS scavengers could exert a protecting effort against endotoxic shock in rodents by hampering TNF-α.

Thus, it has been demonstrated that ozone–oxygen (O_2_O_3_) mixture might play a key role as a microbiocidal agent compared to the rich bactericidal activity of NO, serving as a modulator of several inflammatory processes in vivo [20,21]. O_2_O_3_ exhibits various effects on the immune system, such as the modulation of macrophages’ phagocytic activity, which provides the first-line defense against bacteria and toxins [22].

In this scenario, O_2_O_3_ concentrations should be set to a specific range to ensure safety; however, patients might present a sensation of heaviness at the injection site that spontaneously decreases in a few minutes. On the contrary, other adverse effects might be related to an incorrect administration technique, including vagal crisis, pain, hematoma in the injection site, local infections, and even death [23,24,25].

Moreover, it has been demonstrated that low amounts of ozone increased endogenous antioxidant pathways, entangling glutathione (GSH), superoxide dismutase (SOD) and catalase (CAT), and preparing the host to face ROS-mediated physiopathological circumstances. The ozone, through oxidative preconditioning, protects tissues from ROS-related damage, promoting the antioxidant–prooxidant balance and the concomitant preservation of the cell redox state [26,27,28]. Therefore, we could hypothesize that O_2_O_3_ mixture could enhance proinflammatory cytokine modulation [29].

Musculoskeletal disorders are considered as a common cause of pain and functional disability, predicting a burden that will further increase due to the aging of the population [30,31,32,33,34]. They include all inflammatory and rheumatic diseases affecting the osteoarticular system such as osteoarthritis (OA), but also low back pain and temporomandibular disorders [35,36,37,38,39].

O_2_O_3_ therapy has assumed the role of an adjuvant therapeutic approach in various pathological disorders characterized by chronic inflammatory processes and immune hyper activation, and most musculoskeletal disorders share these two pathophysiological scenarios [40]. In this context, several authors presented a practical function of O_2_O_3_ in the management of low back pain (LBP) with promising perspectives, as a minimally invasive approach, for the conservative therapies of disc herniation or protrusion and in case of failed back surgery syndrome [41,42,43,44,45,46,47]. At the same time, a recent systematic review [48] documented that knee pain could be decreased after O_2_O_3_ intra-articular management in patients affected by knee osteoarthritis (KOA). Likewise, tendon disorders are another conceivable focus for O_2_O_3_ therapy, and a recent randomized controlled trial (RCT) evaluated the usefulness of O_2_O_3_ therapy in patients with shoulder impingement, indicating that it might be assumed an intriguing alternative intervention in case of contraindication to corticosteroids [49]. Moreover, O_2_O_3_ injective treatment reported positive results after O_2_O_3_ injection in patients with lateral chronic epicondylitis not responding to conventional therapy [50]. Lastly, favourable developments have been documented even in rheumatic diseases, where O_2_O_3_ rectal insufflations or autohemotherapy seemed to reveal a profitable safety profile, promoting positive prospective in fibromyalgia [51].

A common denominator of these widespread pathologies is the low-grade inflammatory profile, with a similar serum pattern of inflammatory mediators [52,53,54]. This concept has been recently investigated for the development of more specific and sensitive methods for early diagnosis and follow-up, starting from a detailed and targeted phenotypic characterization of musculoskeletal and temporomandibular disorders [55].

To date, although it has been suggested that O_2_O_3_ therapy could be an effective analgesic treatment, its specific anti-inflammatory effects in terms of serum levels of cytokines modifications are controversial.

**Figure 1:**
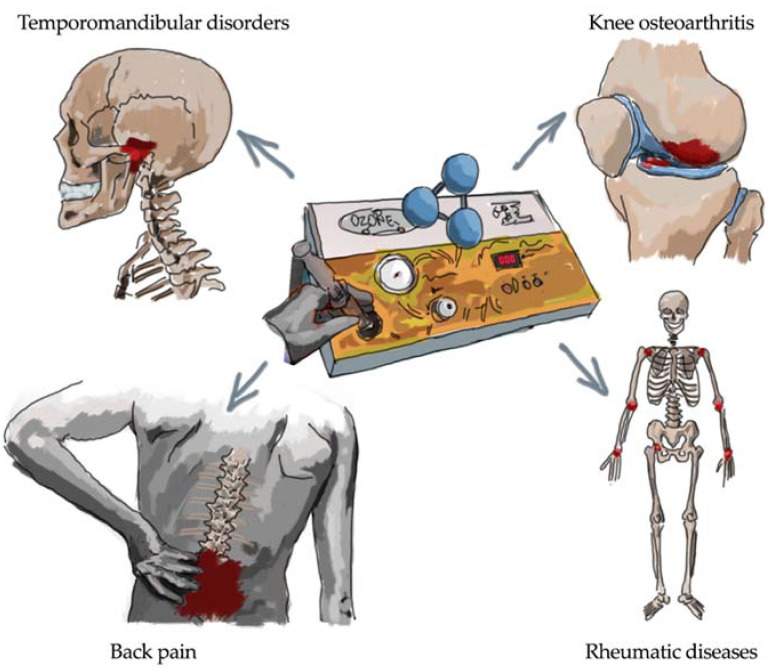
Main clinical targets for oxygen-ozone therapy as anti-inflammatory and analgesic treatment.

Several other musculoskeletal diseases might take advantage of the O_2_O_3_ therapy that is commonly used in the PRM clinical practice. However, only a few papers have investigated the effects of O_2_O_3_ therapy on other musculoskeletal disorders leading to disability (i.e., cervical pain, tendinopathies, and fibromyalgia). Moreover, it should be considered that O_2_O_3_ therapy is commonly used in the clinical practice as anti-inflammatory and analgesic therapeutic option for temporomandibular disorders (TMD) and general musculoskeletal and rheumatic diseases (Figure 1).

Therefore, in the present comprehensive review, we aimed to investigate the state of the art about the effects of O_2_O_3_ therapy on pro-inflammatory cytokines serum levels as a modulator of oxidative stress in patients with TMD and musculoskeletal disorders.

## 2. Oxygen-Ozone as Anti-Inflammatory Therapy

O_3_ is composed of three oxygen atoms with a cyclic structure [56]. It is generated for medical use from pure oxygen which passes through a high voltage gradient (5–13 mV) following the reaction:

3O_2_ + 68,400 cal → 2O_3_

The result is a gas mixture composed of not less than 95% oxygen and not more than 5% O_3_. O_3_ is 1.6 times denser and 10 times more soluble in water than oxygen. It should be considered that O_3_ is the most powerful oxidant after fluorine and persulfate, although it is not a radical molecule. It is an unstable gas that cannot be stored and should be used right away as it has a half-life of 40 min at 20 °C. Despite the heterogeneous and current applications in the medical field, the biochemical effects of O_2_O_3_ are still difficult to understand, even if its properties and chemical characteristics seem to suggest some of its positive clinical effects [40,44,57,58,59].

Like any other gas, O_3_ physically dissolves in pure water according to Henry’s law in relation to temperature, pressure, and ozone concentration. Unlike O_2_, O_3_ reacts immediately with the water present in the tissues. O_3_ reacts with polyunsaturated fatty acids (PUFA), antioxidants such as ascorbic and uric acid, and thiol compounds with -SH groups (cysteine, reduced glutathione-GSH and albumin). Depending on the dose of O_3_ administered, enzymes, carbohydrates, DNA and RNA may also be involved in the process. These compounds undergo oxidation, acting as electron donors [56]. O_3,_ interacting with water and PUFA, present in the tissues, leads to the formation of hydrogen peroxide (H_2_O_2_), a fundamental reactive oxygen species (ROS) which acts as an ozone messenger, and other lipid ozonation products (LOPs) [33,34,35].

H_2_O_2_ is a non-radical oxidant capable of acting as an O_3_ messenger to elicit numerous biological and therapeutic effects. The fact that ROS are always harmful is a concept widely revised in the literature as, in physiological quantities, they are considered mediators of host defense and immune responses. Moreover, they have an extremely short duration (seconds), but by virtue of their reactivity, they could damage cellular components if their generation is not well calibrated. The composition of ROS in the plasma is very rapid and is accompanied by a transient (15–20 min for the recycling of oxidized compounds) and modest decrease in the antioxidant capacity. H_2_O_2_ diffuses very easily from plasma to cells (intracellular gradient 1/10 of the plasma one) and represents an important biological stimulus. In tissues, the moderate oxidative stress of ROS is canceled by endogenous radical scavengers (superoxide dismutase, glutathione peroxidase, catalase, NADPH quinone-oxidoreductase, etc.) [60,61].

An excess of ROS can in fact lead to the formation of toxic compounds such as peroxynitrite (O_5_NOO_2)_ and hypochlorite anion (ClO_2_). Furthermore, the presence of traces of Fe^++^ should be avoided because, in the presence of hydrogen peroxide, they catalyze the formation of the most reactive OH, through the Fenton reaction (hydroxyl radical) [56].

LOPs (lipoperoxides-LOO, alkoxy radicals-LO, lipohydroperoxides-LOOH, iso-prostane and alkenes (4-hydroxy-2,3-transnonenal-HNE and malonyldialdehyde-MDA)) are signal molecules of acute oxidative stress and they cause an upregulation of antioxidant enzymes, such as superoxide dismutase (SOD), GSH-peroxidase (GSH-Px), GSH-reductase (GSH-Rd) and catalase (CAT), which play a key role in antioxidant defense. They also induce oxidative stress proteins, one of which is heme-oxygenase I (HO-1 or HSP-32), which degrades the heme molecule. Being toxic and much more stable in vitro than ROS, they must be generated in very low concentrations and metabolized by GSH-transferase (GSH-Tr) and aldehyde dehydrogenase [56].

Therefore, following the administration of O_3_ the formation of ROS and LOP takes place and, due to their chemical diversity, they act in two different phases. ROS behave as early and short-acting messengers, while on the other hand, LOPs act as late and long-lasting messengers. Thus, they spread in different tissues and bind in small quantities to cell receptors, thus minimizing their toxicity [56].

Small and repeated oxidative stresses might induce the activation of the transcriptional factor mediating nuclear factor-erythroid 2-related factor 2 (Nrf2), a domain involved in the transcription of antioxidant response elements (ARE) and usually bound to protein 1 associated with ECH Kelchlike (Keap-1), thus creating an inactive complex in the intracellular space. A mild oxidative stress can therefore favor the release of Nrf2 from this complex and its migration into the nucleus, where it would favor the transcription of different AREs on the DNA, binding to the Maf protein [62,63].

Therefore, through repeated mild oxidative stresses, O_3_ could induce the upregulation of Nrf2, conditioning human cells to transcribe different AREs, stimulating a better response to pathological radical stress, common in most chronic inflammatory diseases [12].

Several antioxidant enzymes reach a higher level of concentration in response to the production of AREs, such as superoxide dismutase, catalase (CAT), glutathione-transferase (GST), heme oxygenase (HO)-1, heat shock proteins, glutathione peroxidase and quinone-oxidoreductase. These enzymes play a “scavenger role” of free radicals. Based on the redox state of the cell and the amount of O_2_O_3_ administered, we can observe different effects. For example, O_2_O_3_ overexpresses HO-1 or NO-producing 32 kPa heat shock proteins (Hsp34) and, furthermore, Hsp70 expression levels are in turn upregulated by O_2_O_3_, which is related to HO-1. Heme is enzymatically degraded by HO-1 and can be toxic depending on free iron, amount produced and biliverdin. Biliverdin is a nitrosative and oxidative stress neutralizer based on the ability to interact with reactive nitrogen and NO species. The response to thermal shock provides a cytoprotective state during an inflammatory process, aging and neurodegenerative disorders. HO isoforms appear to be regulators of cellular redox homeostasis, functioning as dynamic sensors of its oxidative stress. O_2_O_3_ may play a role in regulating the proinflammatory and anti-inflammatory effects of prostaglandin formation, which is similar in nature to NO [40].

Furthermore, Nrf2 appears to play an important role in the intracellular signaling pathways of inflammation. Indeed, the activation of the Nrf2-antioxidant signal could attenuate a key regulator of the inflammatory response and muscle atrophy (NF-B), and furthermore, the literature suggests that the inflammatory response could be directly down-regulated by suppression of crucial inflammatory mediators and cytokines (IL-6, IL-8 and TNF-a) [64,65,66].

Low doses of O_3_ could therefore play a role in the regulation of prostaglandin synthesis, in the release of bradykinin and in the increase of macrophage and leukocyte secretions. It is widely accepted that pain is a common symptom related to the inflammatory process and O_2_O_3_ therapy could play a key role not only in the management of inflammation, but also in nociceptive perception and modulation. As for the analgesic use of O_2_O_3_, after the administration of O_2_O_3_, an increase in antioxidant molecules (serotonin and endogenous opioids) has been demonstrated, which would induce pain relief by stimulating the antinociceptive pathways [3,6,67,68,69].

Therefore, the effect of O_3_ mimics an acute oxidative stress that, if properly balanced, is not harmful, but is able to elicit positive biological responses and reverse chronic oxidative stress (degenerative process, aging, etc.) This hypothesis about ozone and oxidative stress modulation could be better defined as a “real non-toxic therapeutic shock able to restore homeostasis” [56,70,71].

## 3. Oxygen-Ozone and Back Pain

Little evidence is available in literature about the effect of O_2_O_3_ injections in patients with low back pain due to lumbar disc herniation [72,73]. Although the fluoroscopy or tomography guide requirement could limit the feasibility of this therapy in conventional rehabilitation settings, positive effects were reported in comparison with other interventions such as steroid intraforaminal injection [74]. On the contrary, intramuscular-paravertebral O_2_O_3_ therapy seems to be safe, reliable, and effective to reduce pain in patients affected by LBP not responding to anti-inflammatory/analgesic drugs [75,76].

The O_2_O_3_ might exert its action combining mechanical and anti-inflammatory effects, breaking glycosaminoglycan chains in the nucleus pulposus, decreasing their capability to retain water, thus lowering the size of the herniated position, and allowing to relieve the hernial conflict [22,77]. A reduction in disk volume is the result of all these events. In a study conducted by Andreula et al. [78], five histologic disk specimens were removed during surgical microdiscectomy, providing intradiscal injections of O_3_ at 27 µg/mL, and reporting the nucleus pulposus fibrillary matrix dehydration, regression, and collagen fibers revealing. Parallelly, O_2_O_3_ might also influence the inflammatory cascade by modulating the breakdown of arachidonic acid into prostaglandins and facilitating the fibroblastic action, stimulating the deposition of collagen and the initiation of the repairing process at the tissue level [57].

Around the disc protrusion, inflammatory mediators prompted by granulation tissue are known to attract histiocytes, fibroblasts, and chondrocytes that can produce interleukin-1α (IL-1α), interleukin-6 (Il-6), and TNF-α; these cytokines induce the prostaglandin E2 pathway, which causes pain or increases the sensitivity of the nerve roots to other algogenic substances, such as bradykinin [79]. In vivo, local injection of medical ozone would increase the concentrations of TNF-α, IL1β, and IFN-γ around the disc, suggesting that the contact of medical ozone with the disc damages the extracellular matrix, resulting in shrinkage and decompression of the surrounding neurons. This might proceed probably together with the decrease in lactic acid and inflammatory cytokines, resulting in the decrease of low back pain and sciatica [80].

Furthermore, this disk shrinkage can enhance local microcirculation and increase oxygen supply by decreasing venous stasis caused by disk vessel compression. The O_2_O_3_ therapy might have analgesic and anti-inflammatory effects in treating disk herniation due to the neutralization of proinflammatory cytokines by boosting the surge of antagonists’ release [25].

When a disc degeneration leads to disc herniation, the adjacent nervous system structures, such as the nerve roots, or the dorsal root ganglion can be affected, causing neuropathic pain of mechanical or biochemical origin [81]. Moreover, other spinal structures are damaged, including facet joints, ligaments, and muscles, which can also become pain generators [82]. However, the peripheral sensitization should be avoided by O_2_O_3_ mediators, since recent evidence suggests that ozonized low-density lipoprotein inhibits NFkB and IL-1 receptor-associated kinase 1 (IRAK-1) signaling [83]. At the same time, the oxidation of IL, IL receptors, or nuclear factors might block COX-2 expression [84]. Clinically, Niu et al. showed that low concentrations of medical ozone (20 and 40 μg/mL) can reduce the serum IL-6, IgG, and IgM expression, presenting as analgesic and anti-inflammatory effects; while high concentrations of medical ozone (60 μg/mL) increase the serum IL-6, IgG, and IgM expression, presenting as pain and pro-inflammatory effects. Thus, the medical ozone concentration of 40 μg/mL seemed to report the optimal treatment efficacy [85].

In conclusion, ozone therapy might reduce the autoimmune inflammatory reaction and, consequently, pain due to radiculopathy, after the exposure of the nucleus pulposus to the immune system [22]. Intramuscular O_2_O_3_ therapy is a safe and widely used procedure in the common clinical practice but these results could be only achieved starting from strict eligibility criteria in patient selection and trained and experienced physicians to perform the procedure. Nevertheless, further research must provide evidence for a correct balance between O_2_O_3_ dosage and inflammatory mediators’ expression.

## 4. Oxygen-Ozone and Osteoarthritis

OA is a widespread musculoskeletal disease and a leading cause of chronic disability [86]. Conservative estimates state that up to 240 million people worldwide suffer from it [87]. OA is not merely a degenerative disease, considering that both mechanical and inflammatory factors are attributed to its pathophysiology [88,89,90]. The paradigm of OA is changing from the non-inflammatory theory of “wear and tear” to the hypothesis of “chronic low-grade inflammation” [91,92]. Long-time exposure to chronic low-grade inflammation and imbalance in oxidant-antioxidant systems is involved in OA pathogenesis and progression by compromising the complex network of signaling pathways that regulate cartilage and subchondral bone homeostasis [93,94].

A crucial role in this process might be played by inflammatory cytokines released by chondrocytes (IL-1β, IL-6, IL-8, IL-17, TNF-α, IFN-γ), promoting the catabolism of cartilage and subchondral bone [93,94]. Under normal conditions, these catabolic factors are in equilibrium with anabolic factors that include anti-inflammatory cytokines (IL-4, IL-10) and anabolic cytokines (TGF-β, IGF-1, FGF-18, and PDGF) [95,96,97]. Inflammatory and catabolic factors produce an imbalance that leads a healthy joint to develop OA [97].

As a result, clinical research is looking for immunomodulatory treatments that can act on inflammation to reduce the progression of OA and stimulate the synthesis of anabolic factors [48]. Among these, O_2_O_3_ represents a promising treatment option for its ability to modulate inflammation, promote cartilage growth, and joint repair mechanisms [48,61,98]. O_3_ might influence the modulation of inflammation through different mediators and signaling pathways [4,99,100]. In synovial fluid, O_3_ decreases the production of pro-inflammatory cytokines, particularly IL-6, IL-1β, and TNF-α, which are responsible for cartilage degradation [101]. This effect of ozone has been observed and demonstrated in several studies in animal models of knee OA (KOA), rheumatoid arthritis, and in models of ischemia/reperfusion, e.g., in the reduction of neuropathic pain [3,98,102].

A recent in vivo study on intra-articular O_2_O_3_ injection treatment in patients with KOA has shown that O_3_ is capable of reducing serum levels of IL-6 [91]. This is particularly interesting because IL-6 is produced by IL-1β and TNF-α, two important inflammatory cytokines that appear to play a key role in the initiation and development of OA [103]. IL-1 is responsible for cartilage destruction whereas TNF-α activates the inflammatory process [91]. The authors also demonstrated that O_3_ could be capable of improving serum IGF-1 levels. IGF-1 is a growth factor with important properties in reducing inflammation and stimulating cell growth, differentiation, and tissue repair [104].

Hashemi et al. obtained similar results showing that treatment with intra-articular injections of ozone in patients with KOA induces a significant reduction in serum levels of inflammatory cytokines at 1, 2, and 6 months after the procedure [101]. This result is also greater at 2 and 6 months compared with patients treated with steroid injections. Notably, IL-1b and TNF-α serum levels significantly decreased in the ozone group compared with the steroid group. The authors’ hypothesis is that ozone is likely to have a more stable anti-inflammatory effect than steroids. Although the steroid has a robust anti-inflammatory action against inflammatory cytokines, this effect in cartilage tissue was shorter than ozone. The biochemical findings of this study are also confirmed by the clinical outcomes; in fact, patients treated with ozone demonstrated a more lasting improvement in pain and disability compared to steroid injection.

These results represent in vivo confirmation of previous in vitro experiments focusing on the ability of ozone to reduce serum levels of pro-inflammatory cytokines by stimulating the production of anti-inflammatory cytokines and anabolic chemokines. These intriguing biological effects could be strictly connected to the clinical improvements observed in these patients.

**Figure 2:**
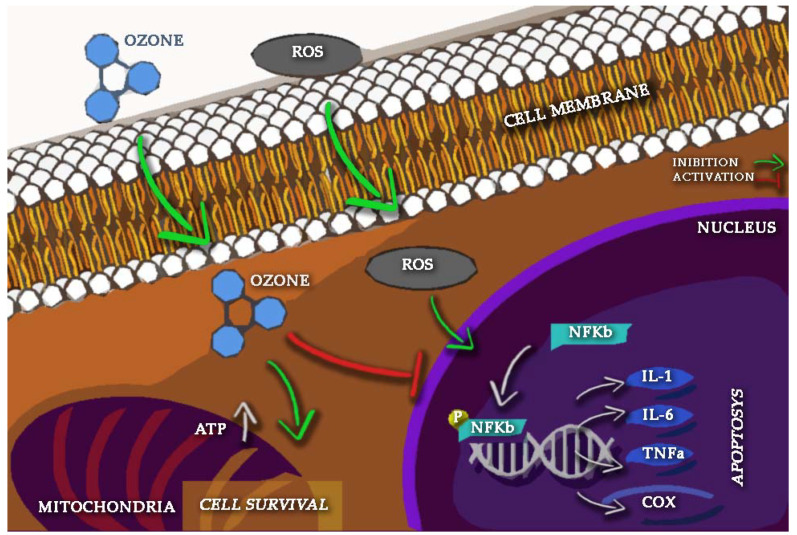
Ozone (O_3_) intracellular and intranuclear pathways involved in inflammation and oxidative stress.

Inflammatory cytokines can also increase the production of ROS which can activate the NF-Кβ pathway leading to accelerating cartilage matrix disintegration and apoptosis [105,106,107,108,109]. Ozone has been observed to decrease the NF-Кβ pathway and enhance the Nrf2 (Nuclear factor erythroid 2-related factor 2) pathway, which is involved in the generation of antioxidant response elements (AREs) such as superoxide dismutase (SOD), catalase (CAT), glutathione peroxidase (GPx), and hemoxygenase-1(HO-1) [110,111]. The activated NF-Кβ pathway could lead to a downstream cascade of other proinflammatory cytokines giving rise to a vicious circle that perpetuates the chronic inflammatory process [112]. Ozone inhibition of NF-Кβ activation can reduce the degradation of the cartilage matrix and initiation of the apoptotic pathway, thus supporting cell survival [108] (Figure 2).

Although injured or damaged articular cartilage remains one of the most difficult tissues to treat [113], it has been recently highlighted that the ozone could provide promising results as a safe and effective treatment in patients with KOA from both a biochemical and clinical perspective [3,114,115,116,117,118,119,120,121].

## 5. Oxygen-Ozone and Rheumatic Diseases

Rheumatoid arthritis (RA) is the most frequent pathology associated with chronic joint inflammation, a genetic degenerative disease that initially affects extremity joints and is characterized by a chronic inflammatory state that distorts and demolishes articular cartilage and expands connective tissue fibrosis, leading to cell destruction and subchondral bone deterioration. It has been estimated that about 1% of the world population suffers from this disorder [122]. O_2_O_3_ therapy effectively decreased inflammation with a down-regulation of pro-inflammatory cytokines and an up-regulation of IL-10 anti-inflammatory cytokine [123]. Rajesh and collaborators investigated the temporal expression of cytokines during the initial phase of an experimental model of arthritic inflammation and revealed that interferon-gamma (IFN-γ) participates in inflammatory process modulation [124]. The O_3_ has also been shown to effectively increase the clinical response of methotrexate (MTX) in patients with rheumatoid arthritis induced by PG/PS. The combination therapy diminishes inflammation through reduction of IL-1B and TNF-α and decreases oxidative stress by reducing hydrogen and preventing damage to proteins and lipids [125].

In this scenario, O_2_O_3_ therapy seems to play a positive role in several inflammatory conditions due to its bacteriostatic, oxidative stress, immune and epigenetic modulation. Compared with topical ozone administration, systematic ozone therapy has apparent advantages in enhancing metabolism, blood hypercoagulability, angiosclerosis, insomnia, and rejuvenation of the body [2,126]. Thus, psoriasis vulgaris is a chronic immune-mediated inflammatory cutaneous disease characterized by red, itchy, and scaly skin patches. Patients typically suffer disfiguration, disability, and associated comorbidities [112]. Zeng et al. indicated that short-term O_2_O_3_ therapy seemed to attenuate psoriatic disease severity lowering the level of blood lipids and up-streaming PPAR-γ level in CD4 T cells, considering that the PPAR-γ expression is commonly reduced in CD4 T cells in psoriasis [127].

Systemic sclerosis (SSc) is an immune-mediated rheumatic disease, characterized by skin and visceral organs fibrosis and vasculopathy [128,129]. Carpal tunnel syndrome (CTS) is one of the most common entrapment disorders in general and the most recurring peripheral nervous system involvement in SSc [130,131]. Elawamy et al. demonstrated that both intracarpal ozone or methylprednisolone reported advantageous impacts upon CTS in people with SSC; nonetheless, ozone relieves pain, enhances the hand functioning, decreases the duration and frequency of Raynaud’s attacks, declines the size of ulceration, and improves median nerve conduction study over the 6-month follow-up [132]. Rascaroli et al. found slight improvements in sensory and motor parameters after ozone therapy, and Bahrami et al. showed improvement of median sensory nerve action potential latency, compared to the pre-treatment level in both groups (one group treated with wrist volar splint alone, the other group treated with ozone injection and splint) [133,134,135,136]. The nature of the fibrotic expression in SSc people with CTS seemed to be significantly associated with gene upstream for Col1 and Col3, TGF-β, and SMAD3 in CTS fibroblasts [137,138]. These studies, focusing on TGF-β signaling inhibition in CTS, reported that therapies targeted for the TGF-β pathway might eventually have utility for the prevention and treatment of CTS, as well as the anti-fibrotic effect of relaxin [137,138].

Gout disease is one of the most frequent causes of inflammatory arthritis in adults and is chronic disorder associated with self-limiting acute gout attacks (gout flare), caused by the accretion of monosodium urate (MSU) depositions in joints and surrounding soft tissue and bursa [139,140,141]. Acute episodes of gout disease are one of the most influential reasons for unfavourable health-related quality of life [139,140,141]. In a rat model, ozone therapy indicated a decrease in the degree of edematous ankle swelling, pro-inflammatory cytokines, lipid peroxidation, the nucleotide-binding oligomerization domain-like receptor containing pyrin domain 3 (NLRP3), procaspase-1, caspase-1, interleukin-1β synovial tissue levels with an enhancement of antioxidant defence system [142]. In other murine models, Bilge et al. demonstrated that ozone therapy raises the levels of antioxidant enzymes, including oxidative shock proteins (hemo-oxygenase-1), Interleukin 4 and Interleukin 10, TGF-β, NO endorphin, adrenocorticotropic hormone (ACTH), and cortisol levels [141].

In conclusion, the positive effect of ozone treatment sustained by its bidirectional regulation of immunity are present also in rheumatic diseases patients. These positive effects could be caused by a O_2_O_3_-related massive production of inflammatory modulation cytokines by immune cells.

## 6. Oxygen-Ozone and Temporomandibular Disorders

Temporomandibular disorders (TMD) represent heterogeneous musculoskeletal disorders, defined as a multifactorial set of signs and symptoms involving masticatory muscles of the stomatognathic system, temporomandibular joint (TMJ), or both [143].

According to the Diagnostic Criteria for TMD (DC/TMD) Axis I, TMD could be divided in muscle disorders (including myofascial pain) or intra-articular disorders (including disc displacement with or without reduction, arthralgia, and arthritis) [144,145].

TMD are the second most common musculoskeletal disorders, affecting approximately 90% of the general population [146,147,148]. Indeed, TMD are the first most common cause of pain of non-dental origin in the maxillofacial region [149], with an incidence rate of 3.9% per annum [150].

Main clinical symptoms are pain and limited jaw range of motion, often accompanied by decrease in the maximal interincisal opening, muscle or joint tenderness on palpation, joint sounds, and otologic complaints (e.g., tinnitus, vertigo, or ear fullness) [151,152]. These signs and symptoms could lead to discomfort or difficulty in performing activities of daily living, such as eating, chewing, talking, swallowing, yawning, causing disability with a significantly reduced quality of life [153,154,155].

The etiology has been accepted as multifactorial, and parafunctional habits, clenching of teeth, grinding, as well as psychosocial issues, including anxiety depression are generally believed to contribute to the development or perpetuation of the pain complaints [156,157,158,159].

In response to this imbalance of the masticatory system, cytokines such as TNFα, IL-1, IL-6 and IL-8 are released within TMJ [160], thus promoting the release of proteinases and stimulating the expression of degrading enzymes and inflammatory mediators; all this mechanism could lead to a TMJ inflammation and bone and cartilage degradation [161]. Other cytokines and metallo-proteinases (MMPs) could be involved in the inflammatory process, including interferon-gamma (IFN-γ), prostaglandin E2 (PGE2), IL-17, MMP-2, MMP-9, aggrecanase-1 and aggrecanase-2 [162,163,164,165].

More in detail, both immune and non-immune cells could release TNF-α (e.g., macrophages, synoviocytes, and neurons associated with the trigeminal ganglion), causing TMJ inflammation and pain in myofascial TMD patients [165]. Ulmner et al. [166] characterized and quantified the synovial tissue cytokines and related the result to the diagnoses of disc displacement with or without reduction. Results of this study showed that bone morphogenetic protein (BMP) type 2 and 4, epidermal growth factor (EGF), eotaxin, granulocyte-colony stimulating factor (G-CSF), IL-1β, IL-7, IL-8, IL-10, macrophage inflammatory protein (MIP) 1β, TNF-α and TNF-β had significantly higher concentrations in patients with disc displacement without reduction. In 2021, Son et al. [167] investigated the relationship between long-term clinical characteristics and different cytokine and autoimmunity levels in young female TMD patients according to pain disability. The subjects included in the study were classified in high and low disability groups, according to the Graded Chronic Pain Scale (GCPS). The authors showed that IL-8 and IgG levels were significantly higher in the high disability group (*p* = 0.047 and 0.005, respectively).

Several conservative treatments have been used for reducing TMD-related pain, including occlusal splint devices [168,169], behavioral therapies [170], manual therapy [171], laser therapy [172], transcutaneous electrical nerve stimulation (TENS) [173], dry needling [173]. In this context, O_2_O_3_ therapy [152,174,175,176] might be considered as a promising new treatment to reduce TMD pain, although the mechanism of action should still be adequately investigated [177]. Probably, O_2_O_3_ might effectively decrease inflammation with a down-regulation of pro-inflammatory cytokines and an up-regulation of IL-10 anti-inflammatory cytokine [123].

In the context of muscle-related TMD, Celakil et al. [178] recently conducted a double-blind randomized clinical trial in order to evaluate the efficacy of ozone therapy compared to placebo. Topical gaseous ozone therapy was applied to the muscles of 20 participants three times per week for 10 min for 2 weeks, with a significantly lower VAS score than placebo group after treatment (*p* = 0.040). Moreover, the pressure pain threshold of the temporal muscle, masseter muscle, and TMJ lateral pole were significantly higher in the ozone group (*p* = 0.035; *p* = 0.007; *p* = 0.012, respectively). The same authors also compared the bio-oxidative ozone therapy to occlusal splints therapy in patients affected by both muscle and articular TMD disorders [135,175,179], showing that both therapies were effective in the improvement of mandibular movements and VAS scores. However, evaluating the effects in terms of PPT measurement of the temporal and masseter, their results indicated that occlusal splint treatment produced better results than ozone application (*p* = 0.046; *p* = 0.024, respectively). In the context of intra-articular TMD, Daif et al. compared the effects of TMJ ozone injections to medication therapy in TMD patients with disc displacement with reduction [180]. In the ozone group, each joint received 2 mL ozone–oxygen mixture (10 μg/mL) injections, 2 times per week for 3 weeks, whereas patients in the second group received nonsteroidal anti-inflammatory drugs and muscle relaxants showing. Results of this study showed that 87% of the patients who received ozone gas injections into the superior joint space either completely recovered (37%) or improved (50%), whereas in the medication group, only 33% of the patients showed an improvement in their clinical dysfunction indexes.

To date, the precise mechanism underpinning the positive effects of O_2_O_3_ therapy in TMD patients are far from being understood. However, O_3_ decreases the production of pro-inflammatory cytokines, particularly IL-6, IL-1β, and TNF-α, which are responsible for cartilage degradation in the synovial fluid [101]. Thus, when injected into a joint capsule, O_2_O_3_ could be able to stimulate the intrinsic fibroblastic joint repairing abilities and to promote new cartilage growth as well as reducing inflammation [180,181]. Therefore, ozone therapy is a not-invasive, fast, and comfortable treatment modality that seems to be effective in the pain management framework in TMD. This could have positive implications for these patients in improving mandibular function, although the precise role of O_2_O_3_ on serum levels of pro-inflammatory cytokines should be better investigated.

## 7. Conclusions

In conclusion, this comprehensive review describes the impact of O_2_O_3_ therapy on serum cytokine levels in different settings and conditions. As previously described, musculoskeletal and rheumatological disorders include several pathological conditions characterized by different and complex therapeutic approaches. In this scenario, O_2_O_3_ therapy remains a promising conservative and minimally invasive intervention that improves pain symptoms and patients’ quality of life. To date, evidence suggests a role of O_2_O_3_ therapy in IL-6 and IL-10 serum level modulation, although the precise epigenetic mechanism remains controversial. Therefore, further high-quality studies are needed to fully understand the molecular, epigenetic, and biochemical effects of O_2_O_3_ and its therapeutic implications.

## Data Availability

Not applicable.
